# Contra‐Thermodynamic Chain‐Walking Isomerization: A Sustainable Tool for Selective Molecular Editing in Green Chemistry

**DOI:** 10.1002/cssc.70724

**Published:** 2026-05-22

**Authors:** Gábor Turczel, Ádám Erdélyi, Márton Nagyházi, Robert Tuba

**Affiliations:** ^1^ Institute of Materials and Environmental Chemistry Research Centre for Natural Sciences Budapest Hungary; ^2^ Research Centre for Biochemical, Environmental and Chemical Engineering, Department of MOL Hydrocarbon and Coal Processing University of Pannonia Veszprém Hungary

**Keywords:** contra‐thermodynamic | chain‐walking | isomerization | tandem reactions | terminal alkenes

## Abstract

Contra‐thermodynamic chain‐walking isomerization has emerged as a powerful strategy in green chemistry for the selective transformation of alkenes and functionalized hydrocarbons under relatively mild (typically below 100°C) conditions. Unlike conventional thermodynamically driven isomerizations that favor the most stable olefin products, contra‐thermodynamic processes enable the migration of double bonds toward less stable, yet more synthetically valuable positions. This directional control allows access to otherwise difficult‐to‐obtain isomers without the need for pre‐functionalized substrates or multistep synthesis. By employing tailored, selective transition‐metal catalysts, light‐driven systems, or redox‐mediated pathways, contra‐thermodynamic chain‐walking can proceed with high atom economy and minimal waste generation. Moreover, this methodology enables even the late‐stage functionalization and valorization of biomass‐derived feedstocks. Continued development of more efficient and recyclable catalytic systems is expected to further expand the environmental and industrial relevance of contra‐thermodynamic chain‐walking isomerization.

## Graphical abstract

Contra‐thermodynamic chain‐walking isomerization enables selective migration of alkenes to less stable yet synthetically valuable terminal position under mild, catalytic conditions. By integrating tailored metal catalysis, photochemical, and redox strategies, this approach affords efficient access to terminal and remote functionalized products with good atom economy, offering promising opportunities for sustainable synthesis and biomass valorization.

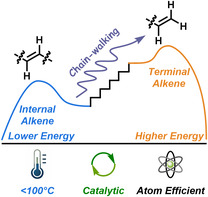

## 1 Introduction

### 1.1 Introduction to Contra‐Thermodynamic Isomerization

**SCHEME 1:**
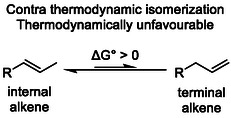
Contra‐thermodynamic isomerization.

Catalytic alkene isomerization represents one of the most atom‐efficient and sustainable transformations in modern organic synthesis, enabling molecular diversification without external reagents, redox steps, or stoichiometric waste generation. Beyond its synthetic utility, this process offers a unique possibility for controlling double bond position and functionality through rearrangements under mild conditions. Notably, terminal alkenes—although typically disfavored under thermodynamic equilibrium—could emerge as reactive intermediates or end products in contra‐thermodynamic isomerizations (Scheme 1).

Isomerization is frequently integrated into tandem, sequential, or domino processes, which are commonly performed in a one‐pot fashion, minimizing purification steps and improving overall efficiency [1, 2, 3, 4, 5, 6]. In many cases, alkene isomerization serves as a dynamic equilibration step that funnels substrates toward thermodynamically favored intermediates, which are then selectively transformed in subsequent reactions. This strategy enhances atom economy and step efficiency, aligning well with the principles of sustainable chemistry. However, when combined with selective trapping reactions or orthogonal catalytic manifolds, isomerization can be harnessed to generate and consume minor, high‐energy species before re‐equilibration occurs. Under such carefully designed conditions, the reaction network can be biased toward the formation of contra‐thermodynamic products, effectively transforming a thermodynamic limitation into a synthetic advantage.

### 1.2 Brief Review of the Most Relevant Isomerization Catalysts

The isomerization of terminal alkenes to their internal counterparts represents a valuable and operationally simple transformation for controlling the positional distribution of functional groups within a given substrate. Although the contra‐thermodynamic alkene isomerization discussed herein constitutes a particularly powerful synthetic strategy, a brief overview of the more common terminal‐to‐internal isomerization is first provided to establish fundamental mechanistic insight into alkene isomerization processes. Notably, there are several selective reactions resulting in terminal alkene products: the Shell higher olefin process (SHOP) [7], the Phillips triolefin process (PTP) [8], and the olefin metathesis in the presence of excess ethylene (ethenolysis) [9, 10] all generate valuable terminal alkenes. It is also worth noting that natural products like allylbenzene derivatives [11] and terpenoids [12] also represent relevant terminal alkene sources. The use of the alkene isomerization reaction is usually represented by either α–β (single‐step) isomerization to get β‐olefins selectively, or otherwise multiple‐bond‐wide isomerization resulting in a variety of internal alkenes. The latter is also known as chain‐walking reaction. In general, when terminal alkenes are readily available, their isomerization to β‐ or other internal positions is relatively straightforward, and a wide range of both stoichiometric and catalytic methodologies can be employed to access the desired products. In a special case, if there is a hydroxy function on the aliphatic hydrocarbon chain, the chain‐walking reaction results in a ketone selectively, as C—C unsaturation irreversibly converts to C—O unsaturation by tautomerization. On the other hand, in the presence of an aromatic ring, the conjugation favors the formation of styrene derivatives.

The selection of an appropriate catalyst or reagent for alkene isomerization largely depends on the structural features and functional group tolerance of the substrate. In stoichiometric approaches, additives are typically acidic or basic in nature. For example, strong mineral acids promote isomerization via protonation of the double bond followed by carbocation formation and subsequent charge migration along the hydrocarbon chain. Such transformations often require elevated temperatures and generally exhibit limited selectivity toward a single positional isomer. Moreover, acidic conditions may induce undesired side reactions, including skeletal rearrangements (e.g., self‐alkylation), and can pose practical challenges due to their corrosive nature toward reaction equipment [13]. Thus, catalytic processes offer reasonable and effective alternatives, also from a green chemistry point of view.

**SCHEME 2:**
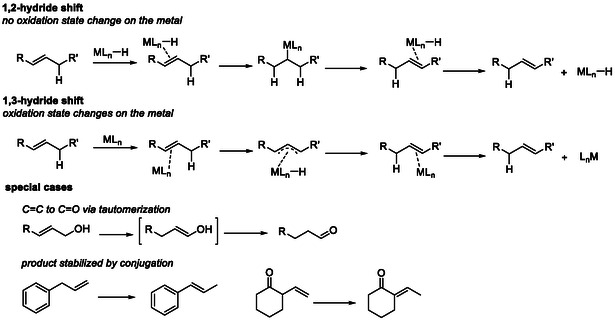
Different mechanisms of alkene isomerization via homogeneous catalysts, along with notable and distinctive aspects related to spontaneous product stabilization.

Catalytic alkene isomerization generally proceeds via two fundamental mechanistic pathways [14]. In the first step, a metal hydride species is added to the C—C bond to form a metal–alkyl intermediate, which subsequently undergoes β‐hydride elimination. This sequence results in an overall formal 1,2‐hydride migration along the carbon chain. Alternatively, the alkene may coordinate to the metal center to generate an η^3^‐allyl intermediate. The corresponding allyl–hydride complex can then revert to a positional isomer of the olefin while regenerating the original metal species, amounting to a formal 1,3‐hydride shift. The formation of allyl intermediates via hydrogen abstraction is often promoted by coordinatively unsaturated or open‐shell metal complexes possessing an accessible unpaired electron. (Scheme 2).

**SCHEME 3:**
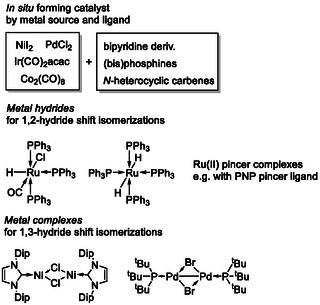
Representative classes of isomerization catalysts.

Homogeneous, metal‐based isomerization catalysts encompass a broad range of structural motifs. In certain cases, the metal precursor does not require a specifically tailored ligand framework [15]. Mechanistic pathways proceeding via a formal 1,2‐hydride shift generally necessitate the presence of a metal–hydride functionality [1, 16]. In contrast, catalytic systems operating through a 1,3‐hydride shift pathway often rely on ligand environments capable of stabilizing uncommon or low‐valent oxidation states (e.g., Ni(I) or Pd(I)), which may undergo facile redox changes during the catalytic cycle (Scheme 3) [17].

### 1.3 Isomerization Tandem Reaction Involving Terminal Alkenes as Intermediates

**SCHEME 4:**
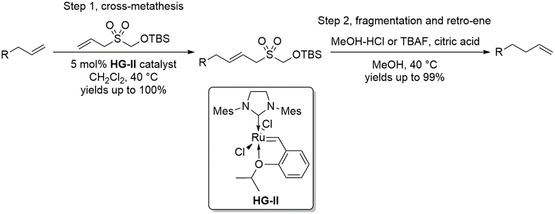
One‐pot terminal alkene homologation. TBS = tert‐butyldimethylsilyl; HG‐II = Second‐generation Hoveyda–Grubbs catalyst.

The synthesis of contra‐thermodynamic compounds, such as terminal alkenes, remains a significant challenge in organic chemistry, where the smart use of reaction sequences enables access to otherwise unfavorable products. Over time, these strategies have become increasingly practical and efficient. For example, one‐carbon homologation of terminal alkenes can be accomplished via a one‐pot protocol that combines olefin metathesis with acid‐mediated fragmentation, followed by a spontaneous retro‐ene reaction (Scheme 4) [18]. In contrast, traditional methodologies typically required four to five discrete synthetic steps, including intermediate purification procedures, to achieve the same overall transformation [19].

The strategic implementation of contra‐thermodynamic isomerization provides an efficient and conceptually powerful approach to the synthesis of terminal alkenes and their derivatives, as it will be demonstrated in the following sections.

## 2 Isomerization‐Hydroboration and Isomerization‐Hydrosilylation

Since thermodynamic alkene isomerization equilibria strongly favor internal alkenes, terminal alkenes are typically present only as minor, contra–thermodynamic components. However, these high‐energy terminal olefin intermediates can be selectively trapped before re‐equilibration occurs through reaction with suitable reagents. In particular, hydroboranes and hydrosilanes provide highly efficient, regio‐ and chemoselective pathways to capture these transient species [20], enabling access to terminally functionalized products from internal alkene feedstocks.

**SCHEME 5:**
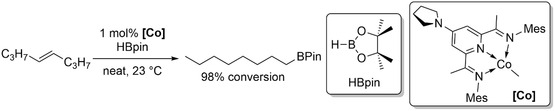
Contra‐thermodynamic tandem isomerization–hydroboration.

Chirik and coworkers developed an efficient tandem isomerization–hydroboration strategy for the synthesis of terminally functionalized boron derivatives (Scheme 5), which serve as valuable intermediates for a wide range of chemical transformations [21].

**SCHEME 6:**
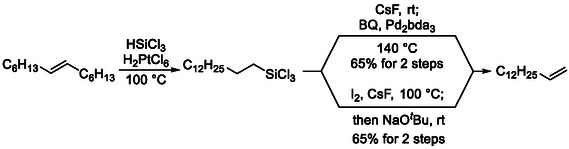
Synthesis of terminal alkenes from internal alkenes via contra‐thermodynamic isomerization–hydrosilylation. BQ = benzoquinone.

**SCHEME 7:**
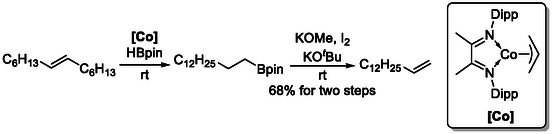
Synthesis of terminal alkenes from internal alkenes via contra‐thermodynamic isomerization–hydroboration.

The Hartwig group reported examples involving both silicon‐ [22, 23] and boronreagents [24], in which catalytic chain‐walking isomerization generated the thermodynamically unfavored terminal alkene (Scheme 6 and 7), which was subsequently trapped by bulky chlorosilanes or borane derivatives to afford the regioselectively functionalized derivatives that were successfully converted into terminal alkenes. Similar reactivity can also be achieved using zirconium‐based isomerization catalysts in combination with hydrosilane reagents [25].

**SCHEME 8:**
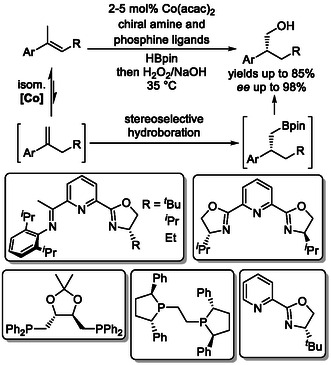
Stereoselective cobalt‐catalyzed contra‐thermodynamic isomerization‐hydroboration. Co(acac)_2 _= cobalt(II) acetylacetonate.

Additionally, chain‐walking isomerization–hydroboration strategies have been successfully applied to the stereoselective synthesis of chiral alcohols (Scheme 8) [26], as well as other enantioenriched derivatives [27]. Optimization of chiral phosphine ligands afforded excellent regioselectivity for boron incorporation at the terminal position in cobalt‐catalyzed isomerizing hydroboration of internal alkenes using Co(acac)_2_, along with high stereoselectivity in the boron addition step, enabling efficient conversion of the intermediates into enantioenriched alcohols.

**SCHEME 9:**
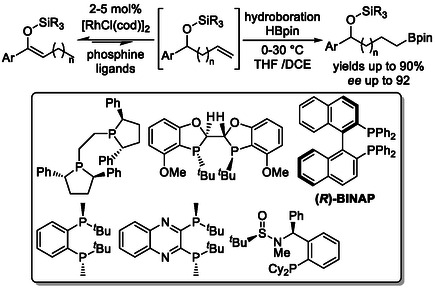
Rhodium‐catalyzed contra‐thermodynamic isomerization‐hydroboration.

The same class of chiral phosphine ligands was successfully applied in a related strategy to synthesize enantioenriched silyl ethers from silyl enol ethers via catalytic chain‐walking isomerization, generating a new stereocenter and enabling terminal functionalization through hydroboration (Scheme 9) [28].

Interestingly, in addition to H–B reagents, B–B derivatives can also be employed in isomerization–borylation reactions, thereby expanding the scope of accessible boron‐functionalized products, including diborated compounds [29].

## 3 Isomerization–Hydrocarboxylation

**SCHEME 10:**
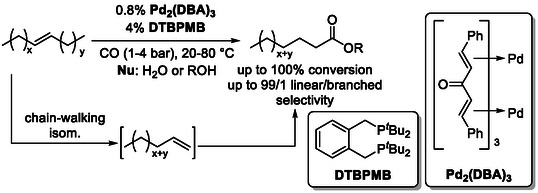
General depiction of the isomerization‐hydrocarboxylation of internal alkenes.

The isomerization–hydroxycarbonylation (alkoxycarbonylation) reaction has a history spanning more than 25 years [30]. Palladium complexes bearing bi‐ or tridentate phosphine ligands efficiently catalyze both chain‐walking isomerization of internal double bonds and subsequent addition of carbon monoxide and alcohol nucleophiles to the contra‐thermodynamic terminal alkenes formed in situ (Scheme 10) [31]. This tandem process enables the direct conversion of internal alkenes into terminally functionalized carboxylic acids or esters with high regioselectivity, providing a powerful and atom‐economical strategy for the valorization of simple olefin feedstocks.

**SCHEME 11:**
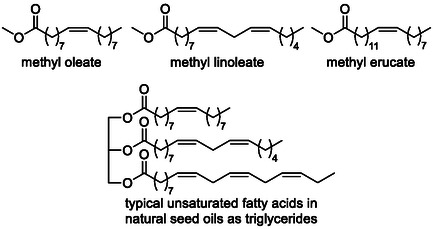
Renewable feedstock for isomerization‐carboxylation reactions.

One of the most promising feedstocks for isomerization–hydroxycarbonylation reactions is renewable seed oils and their long‐chain fatty acid derivatives (Scheme 11), aligning this chemistry closely with the principles of sustainable and green chemistry. The valorization of these abundant, bio‐based resources has been a major driving force beyond catalyst development in this area, particularly for achieving high regioselectivity and functional group tolerance. The literature reports successful examples involving both direct conversion of raw oils [32, 33, 34] (via glyceride substrates) and transformations of pre‐esterified fatty acid derivatives [35, 36, 37, 38, 39, 40, 41], most notably methyl oleate, methyl linoleate, and methyl erucate.

**SCHEME 12:**
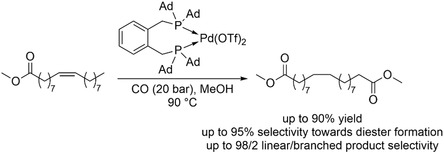
Conversion of methyl oleate to *α*,*ω*‐diester via isomerization‐alkoxycarbonylation. Ad = adamantyl.

Yields of up to 90% and excellent selectivity (>95%) toward the linear *α*,*ω*‐diester were achieved in the conversion of methyl oleate using adamantyl‐substituted diphosphine palladium complexes (Scheme 12) [39].

Rigorous ligand development in this field has extended well beyond empirical substrate screening and catalyst optimization. Computational studies and theoretical calculations have been extensively employed to elucidate the underlying reaction mechanisms, identify key intermediates, and rationalize regioselectivity trends. These insights have, in turn, guided the rational design of improved ligand architectures, enabling more efficient and selective catalytic systems [42]. The development of this concept has already progressed beyond proof‐of‐principle studies, with reports demonstrating scalability to the 100 g level [41]. In parallel, efficient downstream processing strategies, including product diester purification by crystallization and catalyst recovery and reuse, have been extensively investigated [35]. These advances highlight the growing maturity of the methodology and its potential for practical and industrial implementation. Further transformations of the *α*,*ω*‐diester products include derivatization [36] and polymerization [36, 40, 41] to yield polyesters, highlighting their potential as renewable feedstocks for sustainable materials utilization.

**SCHEME 13:**
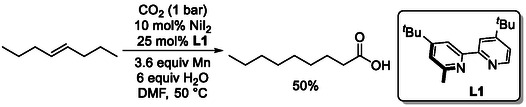
Isomerization‐hydrocarboxylation of internal alkenes utilizing carbon dioxide as carbonyl source.

While most reported examples employ palladium catalysts, this versatile transformation can also be achieved using nickel (Scheme 13). In these systems, stoichiometric manganese, alongside water, is required not only as a reducing agent but also for the generation of the catalytically active species, as demonstrated by Martin and coworkers [43]. In contrast to earlier examples, this process utilizes carbon dioxide rather than carbon monoxide as the carbonyl source.

## 4 Isomerization–Hydroformylation

**SCHEME 14:**
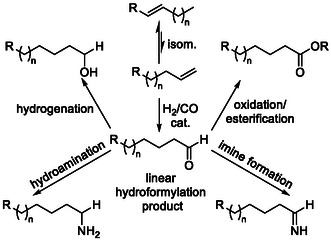
General depiction of the isomerization‐hydroformylation tandem reaction.

By far the most extensively studied approach to contra‐thermodynamic product formation via isomerization is hydroformylation. This tandem process enables the synthesis of terminally functionalized aldehydes, alcohols, amines, and a wide range of related compounds (Scheme 14) from internal alkenes through the selective reaction of thermodynamically unfavored olefin intermediates with H_2_/CO.

Both the isomerization and hydroformylation steps can be catalyzed by a wide range of transition metals [44], including Rh [45, 46, 47, 48, 49], Pd [50], Co [51], Ru [52], and even Fe [53]. However, this section focuses on examples in which renewable substrates are the most prominently employed feedstocks and subsequently highlights related tandem processes, such as isomerization–hydroformylation–hydrogenation reactions.

**SCHEME 15:**
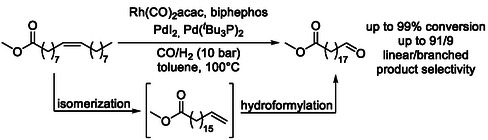
Isomerization‐hydroformylation of methyl oleate.

**SCHEME 16:**
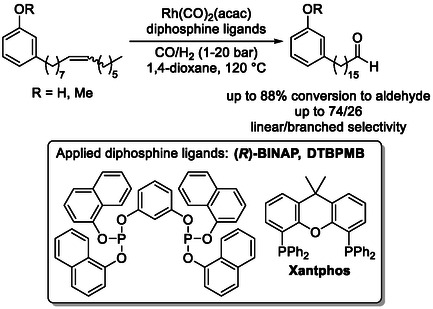
Isomerization‐hydroformylation of cashew nut shell liquid‐derived molecules. For (*R*)‐BINAP, see Scheme 9; for DTBPMB, see Scheme 10.

Sustainable examples include isomerization–hydroformylation tandem reactions of plant‐based feedstocks, particularly linear fatty acid derivatives such as methyl oleate (Scheme 15) [54, 55, 56, 57], which serve as readily available and structurally simple renewable substrates. In addition, non‐edible plant‐derived oils, including cashew nut shell liquid (Scheme 16) [58], which contains aromatic components, have also been successfully employed, highlighting the versatility of this methodology for the valorization of diverse biomass resources.

**SCHEME 17:**
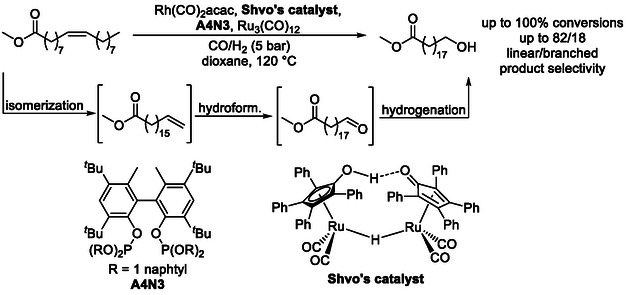
Isomerization followed by hydroformylation and hydrogenation.

Expanding this reactivity through the incorporation of additional tandem steps enables access to a broad range of contra‐thermodynamic products from internal alkenes. For example, coupling isomerization–hydroformylation with hydrogenation [59] allows direct conversion of sustainable feedstocks such as methyl oleate into valuable linear *α*,*ω*‐ester–alcohols up to 82:18 linear : branched selectivity (Scheme 17) [55].

**SCHEME 18:**
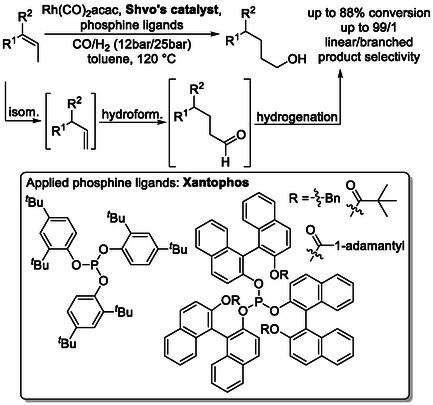
Isomerization followed by hydroformylation and hydrogenation. For Shvo's catalyst, see Scheme 17; for Xantphos, see Scheme 16.

The use of BINAP‐derived phosphine ligands in related systems affords yields of up to 94% and linear selectivities as high as 99% for small internal alkenes (Scheme 18), demonstrating the power of ligand effect in these transformations [60]. These results suggest that, with continued ligand screening and development, such processes could reliably deliver contra‐thermodynamic products even from sustainable and structurally complex feedstocks.

**SCHEME 19:**
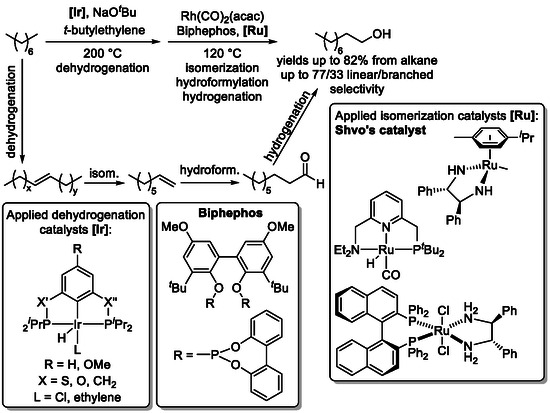
Transformation of alkanes to terminal alcohols via a dehydrogenation–isomerization–hydroformylation–hydrogenation tandem reaction. For Shvo's catalyst, see Scheme 17.

Incorporation of an additional dehydrogenation step further expands this strategy to include alkanes as starting materials. Initial dehydrogenation generates an alkene intermediate, which undergoes isomerization–hydroformylation to form a contra‐thermodynamic aldehyde, followed by hydrogenation to afford terminal alcohols directly from simple alkanes (Scheme 19) [61].

**SCHEME 20:**
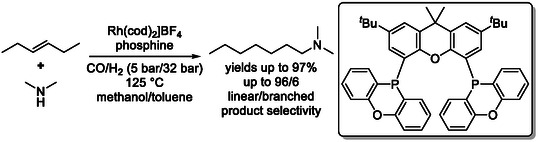
Isomerization‐hydroamination of 3‐hexene.

Terminal amines can also be accessed from internal alkenes through tandem isomerization–hydroformylation–hydroamination sequences [62, 63, 64]. These reactions proceed analogously to the previously discussed systems and typically employ bidentate phosphine ligands in combination with a single metal catalyst, most commonly rhodium complexes, to carry out all transformation steps within one catalytic cycle (Scheme 20). This integrated approach enables efficient conversion of internal olefins into valuable nitrogen‐containing products with high regioselectivity.

An unusual variant of alkene hydroformylation has also been applied to isomerizing hydroformylation, employing N‐formyl saccharin and formic acid as H_2_ and CO surrogates to synthesize terminal aldehydes from internal alkenes in a palladium‐catalyzed process [50].

## 5 Additional Methods

### 5.1 Contra–Thermodynamic Chain–Walking Isomerization Involving Photochemical Approaches

**SCHEME 21:**
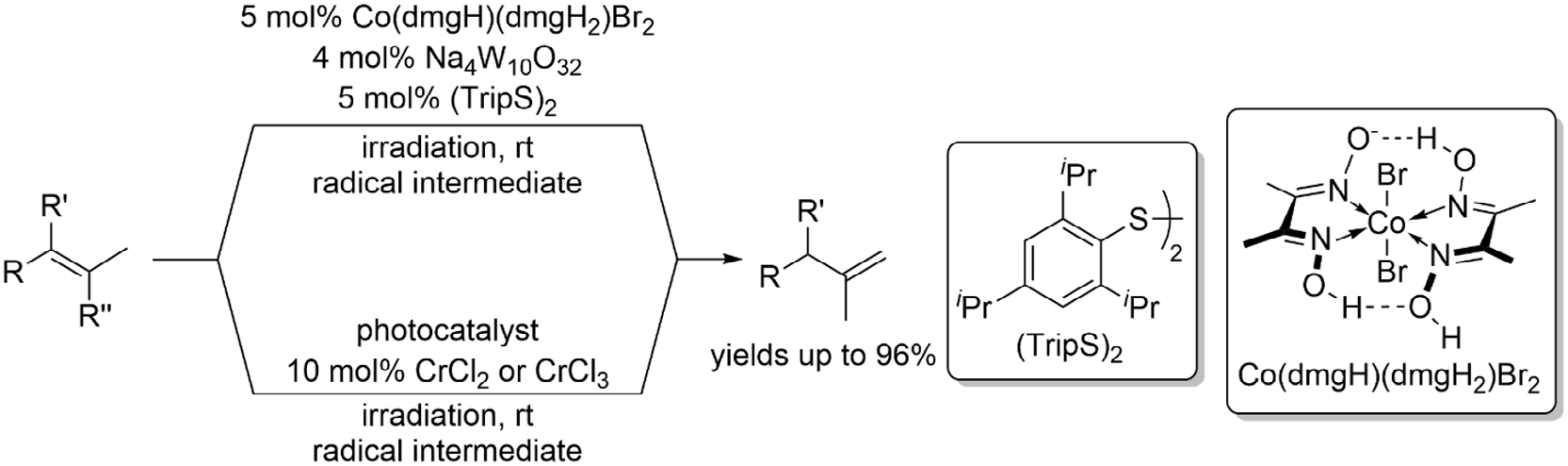
Chromium‐ and cobalt‐catalyzed contra‐thermodynamic isomerization.

Photochemical methods involving Co‐ [65] and Cr‐based [66] catalysts have been successfully applied to convert internal alkenes into thermodynamically unfavored terminal alkenes (Scheme 21). In both systems, β‐olefins were typically transformed into the corresponding α‐olefins in good yields. However, for the Cr‐based catalyst, double‐bond migration was generally limited to a single positional shift. In the case of the Co‐based system, one‐step migration proceeded in good to excellent yields, but only a single example of two‐step double‐bond migration was reported, and the doubly migrated product was obtained only in minor amounts. These observations highlight the limitations of these photochemical protocols when extended to long‐chain alkene substrates requiring multiple migration steps.

### 5.2 Isomerization–Hydrohalogenation

**SCHEME 22:**
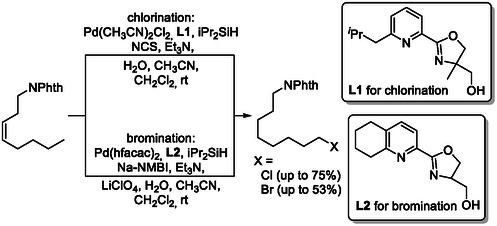
Contra‐thermodynamic tandem isomerization–hydrohalogenation reaction. NPhth = Phthalimide; hfacac = hexafluoroacetylacetonate.

Contra‐thermodynamic tandem isomerization–hydrohalogenation reactions have also been successfully developed, employing palladium catalysts to access terminal chlorides and bromides from internal alkenes (Scheme 22) [67]. This methodology was applied to linear octene isomer mixtures, affording the corresponding terminal chlorides and bromides in good to moderate yields (75% for chlorides and 53% for bromides, respectively). These results demonstrate the potential of this approach to transform inexpensive refinery feedstocks into value‐added terminally functionalized products.

### 5.3 Isomerization–Hydrocyanation

**SCHEME 23:**
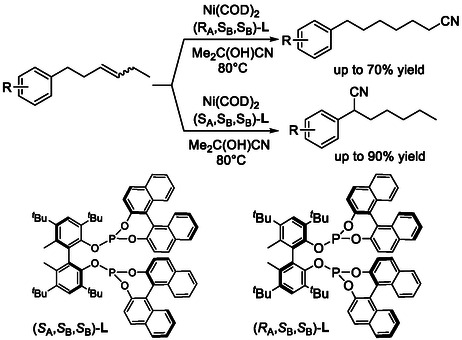
Isomerization–hydrocyanation of internal alkenes, highlighting catalyst‐dependent product selectivity.

Following extensive ligand screening, nickel‐catalyzed hydrocyanation can be directed to produce either the thermodynamically favored or the contra‐thermodynamic product from an internal alkene by employing different diastereomers of BIPOL‐phosphine–BINOL ligands (Scheme 23) [68]. In this regiodivergent system, subtle changes in ligand geometry profoundly influence the migration pathway and the site of C—CN bond formation, allowing the same internal alkene substrate to be selectively transformed into either linear or branched nitrile products with high regioselectivity and good yields. Mechanistic studies indicate that these subtle structural differences modulate both electronic and steric interactions at the nickel center, thereby altering the relative energetics of chain‐walking intermediates and directing the catalyst toward distinct hydrocyanation outcomes.

### 5.4 Isomerization–Hydrooxygenation

**SCHEME 24:**
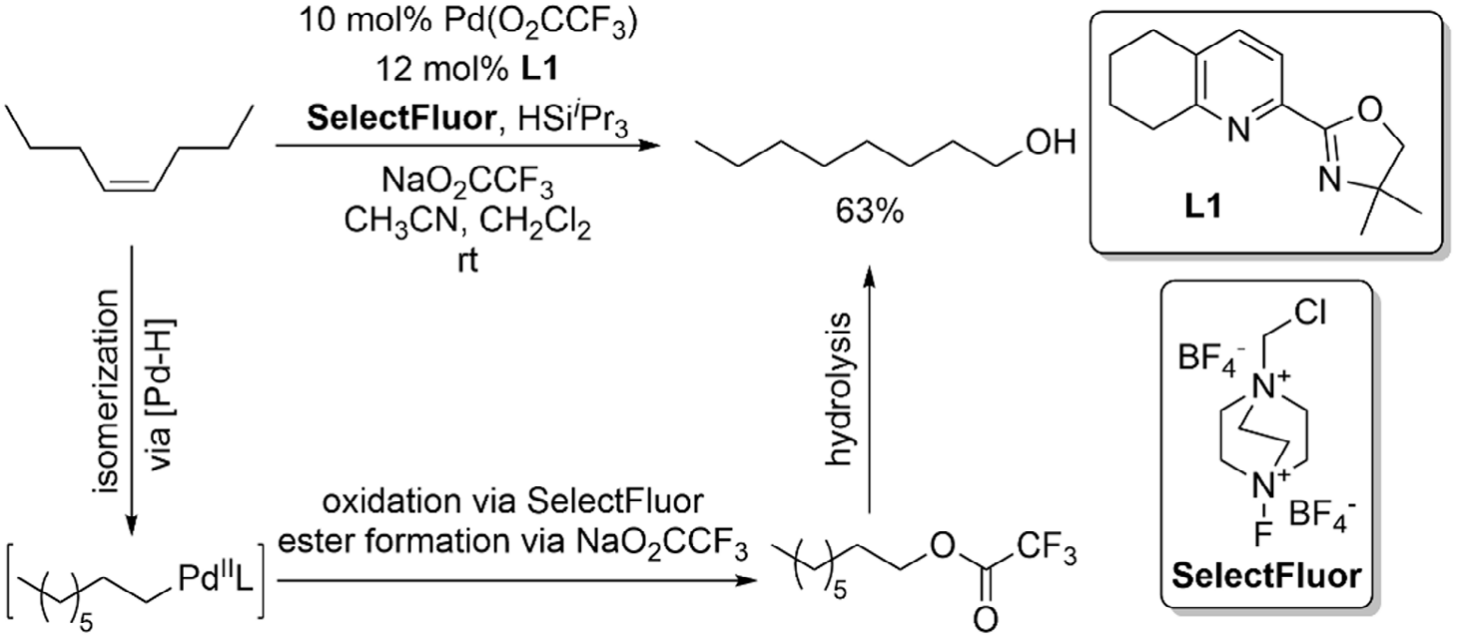
Contra‐thermodynamic tandem isomerization–hydrooxygenation reaction.

A one‐pot isomerization–hydrooxygenation tandem reaction was carried out using a palladium catalyst to synthesize terminal alcohols from internal alkenes (Scheme 24) [69]. In this transformation, the palladium mediates both chain‐walking isomerization and subsequent oxygen incorporation, enabling selective functionalization of the transient, contra‐thermodynamic terminal alkene intermediate. The reaction efficiently delivers the corresponding linear alcohols with high chemo‐ and regioselectivity, demonstrates broad substrate scope, and exhibits excellent tolerance toward diverse functional groups, making it a versatile and practical approach for accessing terminal alcohols from readily available terminal and internal alkenes.

### 5.5 Isomerization–Amidation

**SCHEME 25:**
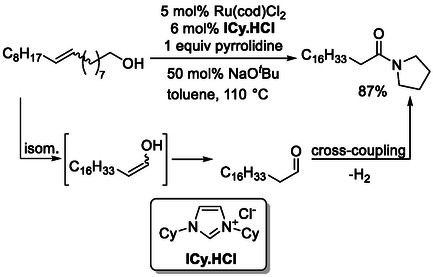
Carbene‐catalyzed isomerization‐amidation involving unsaturated alcohol substrates.

Long‐range isomerization of internal alkenes bearing alcohol groups, followed by ruthenium‐catalyzed cross‐coupling in the presence of amines, affords terminal amides (Scheme 25) [70]. In this redox‐neutral tandem process, the ruthenium catalyst mediates both the remote chain‐walking isomerization and the subsequent C—N bond‐forming transformation. The active carbene ligand is generated in situ from the corresponding iminium precursor salt and is essential for catalytic activity. The presence of base is particularly crucial, as it not only promotes deprotonation of the precursor salt to generate the catalytically active carbene species but also facilitates activation of the secondary amine coupling partner. In the absence of base, the catalytic cycle cannot be initiated, and no product formation is observed.

**SCHEME 26:**
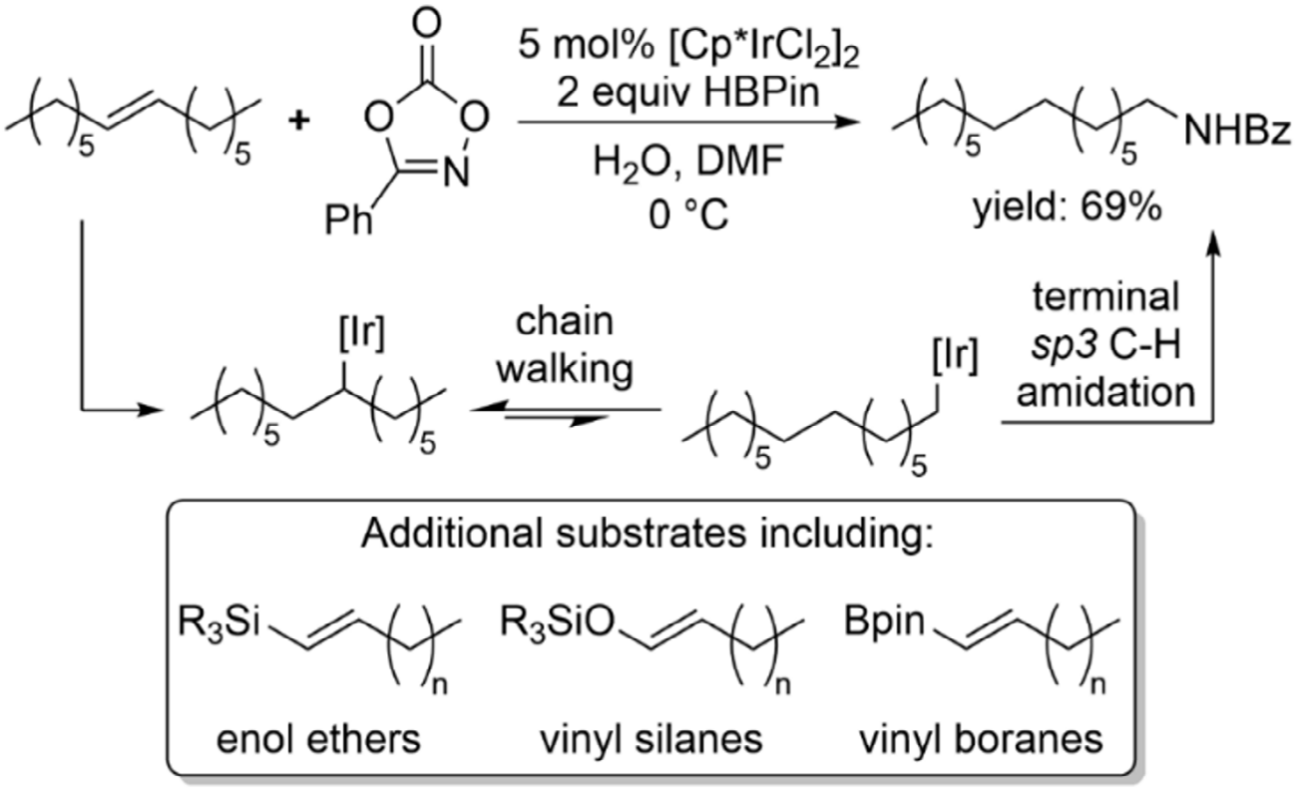
Iridium‐catalyzed contra‐thermodynamic isomerization‐amidation conversion of internal alkenes to terminal amides with dioxazolone reagent.

Iridium‐catalyzed chain‐walking isomerization paired with an appropriate dioxazolone reagent affords terminal amides from internal alkenes (Scheme 26) [71]. In this transformation, the iridium catalyst mediates both the migratory isomerization of the internal double bond and the subsequent C(sp^3^)—H amidation at the terminal position. The substrate scope included not only simple dialkyl‐substituted alkenes but also heteroatom‐substituted olefins, like enol ethers, vinyl silanes, and boron‐containing alkenes.

### 5.6 Isomerization–Metathesis

**SCHEME 27:**
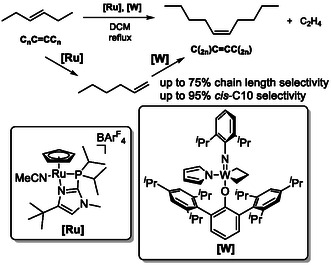
Selective conversion of *trans*‐hexene to *cis*‐decene via isomerization metathesis, involving terminal alkenes as intermediates.

Isomerization metathesis (ISOMET) can also be employed in a way that the highly selective tungsten‐based metathesis catalyst reacts exclusively with the terminal (or *Z*‐internal) alkenes, while chain‐walking isomerization proceeds continuously via *E* alkenes (Scheme 27). This tandem process enables the selective conversion of internal *E* alkenes (C_*n*_C—CC_*n*_) into *Z* alkenes (C_(2n)_C—CC_(2n)_) via transient, contra‐thermodynamic terminal olefin intermediates (C_(2n−2)_CH—CH_2_) [72]. Because the applied Grotjahn‐type "alkene zipper" catalyst generates selectively *E* alkenes throughout the chain‐walking process, the highly *Z*‐ and terminal‐selective metathesis catalyst avoids the formation of multiple homologous side products, that are typically observed in conventional isomerization–metathesis sequences.

### 5.7 Isomerization–Alkylation

**SCHEME 28:**
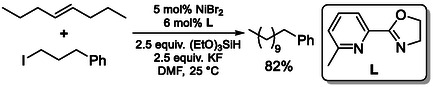
C—C bond formation via isomerization‐alkylation.

The conversion of internal alkenes through contra‐thermodynamic isomerization coupled with alkylation using alkyl halides has emerged as a versatile strategy for remote C—C bond formation (Scheme 28) [73, 74, 75]. These transformations are most commonly enabled by nickel‐based catalytic systems, typically generated from Ni(II) halide precursors (e.g., NiBr_2_ or NiI_2_) in combination with bidentate nitrogen ligands such as bipyridine, pyridine–oxazoline, or related heterocyclic frameworks. Mechanistically, the reaction is initiated by formation of a Ni—H species, typically generated in situ through interaction of the nickel precursor with a hydrosilane. This Ni—H intermediate undergoes hydrometallation with the internal alkene to form an alkyl–nickel species, which subsequently engages in chain walking via iterative β‐hydride elimination and reinsertion steps. Through this process, the metal center migrates along the carbon chain, enabling access to less substituted, alkyl–nickel intermediates. At the terminal position, the resulting alkyl–nickel species undergoes cross‐coupling with an alkyl halide, leading to the formation of terminally functionalized products. Notably, the presence of KF is essential for catalytic turnover. Replacement or omission of KF (e.g., substitution with CsF) results in complete loss of reactivity, suggesting a critical role in facilitating anion exchange processes, likely by converting less reactive nickel iodide species formed after reductive elimination into more catalytically reactive intermediates.

**SCHEME 29:**
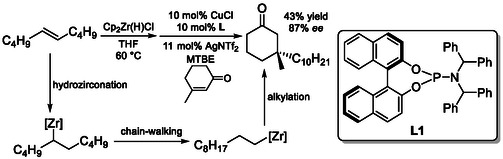
Isomerization‐alkylation utilizing Zr/Cu system.

Organozirconium reagents also exhibit the ability to promote isomerization–alkylation sequences [76]. In these systems, not only alkyl halides but also α,β‐unsaturated ketones can serve as electrophilic coupling partners. In a representative example (Scheme 29), zirconium enabled chain‐walking generates a terminal organozirconium intermediate, which, in combination with copper(I) catalysis, undergoes conjugate addition to an unsaturated ketone [77]. This tandem process ultimately furnishes substituted ketones, highlighting the versatility of organozirconium intermediates in enabling contra‐thermodynamic functionalization pathways.

### 5.8 Isomerization–Hydroarylation

**SCHEME 30:**
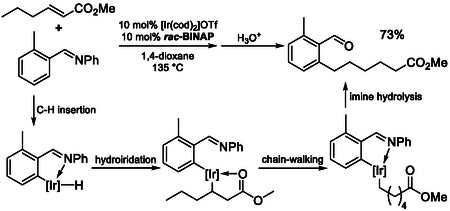
Isomerization‐hydroarylation utilizing iridium‐BINAP systems.

Isomerization–hydroarylation represents a tandem strategy in which alkene chain walking is coupled with C—H activation‐based arylation to achieve remote C—C bond formation (Scheme 30). This reactivity is typically enabled by iridium [78, 79, 80], rhodium [81, 82] or nickel [83, 84] catalysts. The transition metal first activates the arene or heteroarene via C—H insertion, generating an aryl–metal species, which subsequently undergoes hydrometallation across the internal alkene. The resulting alkyl–metal intermediate can then engage in reversible chain‐walking isomerization through subsequent hydride insertion and β‐hydride elimination steps, allowing migration along the carbon backbone and formation of terminal intermediates. Finally, reductive elimination furnishes the C—C bond, delivering the hydroarylated product.

### 5.9 Reactive Distillation

**SCHEME 31:**
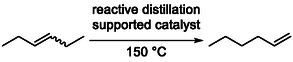
Production of terminal alkenes from internal alkenes via reactive distillation.

A smart strategy for the synthesis of thermodynamically unfavored terminal alkenes involves the reactive distillation of internal alkenes, as described in a patent. In this process, a supported isomerization catalyst is integrated into a distillation column, enabling catalytic rearrangement and separation to occur simultaneously (Scheme 31). Internal alkene isomers are converted into 1‐alkenes, which typically have lower boiling points and are selectively removed overhead, thereby driving the equilibrium toward product formation [85].

## 6 Summary and Outlook

Contra‐thermodynamic chain‐walking enables the formation of α‐olefins and their derivatives from internal olefin feeds. Selective trapping of these terminal alkene intermediates shifts the reaction network toward products that are thermodynamically disfavored under equilibrium conditions. The broad range of compatible trapping reagents allows access to a diverse family of terminally functionalized compounds, significantly expanding the synthetic utility of simple olefin feedstocks. Appropriate catalyst choice can ensure excellent regio‐ and stereocontrol; however, maintaining orthogonality between catalysts remains a major consideration in tandem catalytic systems. As an atom‐economic transformation, alkene isomerization aligns well with the principles of green chemistry and offers a sustainable approach to the valorization of renewable resources such as plant oils and fatty acid esters. Although many of the most developed systems rely on noble metal catalysts, ongoing efforts toward the development of efficient earth‐abundant metal alternatives are expected to further enhance the economic and environmental viability of the process.

## Funding

This study was supported by Nemzeti Kutatási Fejlesztési és Innovációs Hivatal (2024‐1.2.3‐HU‐RIZONT‐2024‐00074; OTKA K‐147172).

## References

[1] D. Fiorito , S. Scaringi , and C. Mazet , “Transition Metal‐Catalyzed Alkene Isomerization as an Enabling Technology in Tandem, Sequential and Domino Processes,” Chemical Society Reviews 50 (2021): 1391–1406. 33295370 10.1039/d0cs00449a

[2] S. Scaringi , and Clément Mazet , “Transition Metal‐Catalyzed (remote) Deconjugative Isomerization of α,β‐Unsaturated Carbonyls,” Tetrahedron Letters 96 (2022): 153756.

[3] C. Romano and R. Martin , “Ni‐Catalysed Remote C(sp3)–H Functionalization Using Chain‐Walking Strategies,” Nature Reviews Chemistry 8 (2024): 833–850. 39354168 10.1038/s41570-024-00649-4

[4] S. Ghosh , S. Patel , and I. Chatterjee , “Chain‐walking Reactions of Transition Metals for Remote C–H Bond Functionalization of Olefinic Substrates,” Chemical Communications 57 (2021): 11110–11130. 34611681 10.1039/d1cc04370f

[5] H. Sommer , F. Juliá‐Hernández , R. Martin , and I. Marek , “Walking Metals for Remote Functionalization,” ACS Central Science 4 (2018): 153–165. 29532015 10.1021/acscentsci.8b00005 PMC5833012

[6] Y. Wang , Y. He , and S. Zhu , “NiH‐Catalyzed Functionalization of Remote and Proximal Olefins: New Reactions and Innovative Strategies,” Accounts of Chemical Research 55 (2022): 3519–3536. 36350093 10.1021/acs.accounts.2c00628

[7] W. Keim , “Oligomerization of Ethylene to α‐Olefins: Discovery and Development of the Shell Higher Olefin Process (SHOP),” Angewandte Chemie International Edition 52 (2013): 12492–12496. 24249549 10.1002/anie.201305308

[8] R. L. Banks and G. C. Bailey , “Olefin disproportionation. A new catalytic process,” Industrial & Engineering Chemistry Product Research and Development 3(1964):170–173.

[9] J. Bidange , Cédric Fischmeister , and C. Bruneau , “Ethenolysis: A Green Catalytic Tool to Cleave Carbon–Carbon Double Bonds,” Chemistry – A European Journal 22 (2016): 12226–12244. 27359344 10.1002/chem.201601052

[10] J. Spekreijse , J. P. M. Sanders , J. H. Bitter , and E. L. Scott , “The Future of Ethenolysis in Biobased Chemistry,” ChemSusChem 10 (2017): 470–482. 27860333 10.1002/cssc.201601256

[11] M. Hassam , A. Taher , G. E. Arnott , I. R. Green , and W. A. L. van Otterlo , “Isomerization of Allylbenzenes,” Chemical Reviews 115 (2015): 5462–5569. 25993416 10.1021/acs.chemrev.5b00052

[12] J. D. Tibbetts and S. D. Bull , “Dimethyl sulfide facilitates acid catalysed ring opening of the bicyclic monoterpenes in crude sulfate turpentine to afford *p* ‐menthadienes in good yield,” Green Chemistry 23 (2021): 597–610.

[13] H. E. Basbug Alhan , G. R. Jones , and E. Harth , “Branching Regulation in Olefin Polymerization via Lewis Acid Triggered Isomerization of Monomers,” Angewandte Chemie 132 (2020): 4773–4779. 10.1002/anie.201914742 31881118

[14] S. Biswas , “Mechanistic Understanding of Transition‐Metal‐Catalyzed Olefin Isomerization: Metal‐Hydride Insertion‐Elimination vs. π‐Allyl Pathways,” Comments on Inorganic Chemistry 35 (2015):300–330.

[15] A. Vasseur , J. Bruffaerts , and I. Marek , “Remote Functionalization through Alkene Isomerization,” Nature Chemistry 8 (2016): 209–219. 10.1038/nchem.2445 26892551

[16] S. Sanz‐Navarro , M. Mon , A. Doménech‐Carbó , et al., “Parts–per–million of Ruthenium Catalyze the Selective Chain–walking Reaction of Terminal Alkenes,” Nature Communications 13 (2022): 2831. 10.1038/s41467-022-30320-9 PMC9123009 35595741

[17] A. Kapat , T. Sperger , S. Guven , and F. Schoenebeck , “E ‐Olefins through intramolecular radical relocation,“Science 363 (2019): 391–396. 30679370 10.1126/science.aav1610

[18] M. C. Grocott and M. J. Gaunt , “One‐Carbon Homologation of Alkenes,” Nature 643 (2025): 130–138. 40393510 10.1038/s41586-025-09159-9 PMC12221989

[19] H. M. Organ , M. A. Holmes , J. M. Pisano , et al., “Novel Derivatives at the C21 Position of the FK‐506 Macrocycle,” Bioorganic & Medicinal Chemistry Letters 3 (1993): 657–662.

[20] J. V. Obligacion and P. J. Chirik , “Earth‐Abundant Transition Metal Catalysts for Alkene Hydrosilylation and Hydroboration,” Nature Reviews Chemistry 2 (2018): 15–34. 10.1038/s41570-018-0001-2 PMC6365001 30740530

[21] J. V. Obligacion and P. J. Chirik , “Bis(imino)pyridine Cobalt‐Catalyzed Alkene Isomerization–Hydroboration: A Strategy for Remote Hydrofunctionalization with Terminal Selectivity,” Journal of the American Chemical Society 135 (2013): 19107–19110. 24328236 10.1021/ja4108148

[22] S. Hanna , T. W. Butcher , and J. F. Hartwig , “Contra‐thermodynamic Olefin Isomerization by Chain‐Walking Hydrofunctionalization and Formal Retro‐hydrofunctionalization,” Organic Letters 21 (2019): 7129–7133. 31424215 10.1021/acs.orglett.9b02695

[23] S. Hanna , T. Wills , T. W. Butcher , and J. F. Hartwig , “Palladium‐Catalyzed Oxidative Dehydrosilylation for Contra‐Thermodynamic Olefin Isomerization,” ACS Catalysis 10 (2020): 8736–8741. 40756329 10.1021/acscatal.0c02697 PMC12316060

[24] S. Hanna , B. Bloomer , N. R. Ciccia , T. W. Butcher , R. J. Conk , and J. F. Hartwig , “Contra‐Thermodynamic Olefin Isomerization by Chain‐Walking Hydroboration and Dehydroboration,” Organic Letters 24 (2022): 1005–1010. 35080409 10.1021/acs.orglett.1c03124 PMC8931855

[25] O. A. Luongo , M. Lemmerer , S. L. Albers , and J. Streuff , “Methoxide‐Enabled Zirconium‐Catalyzed Migratory Alkene Hydrosilylation,” Angewandte Chemie International Edition 63 (2024): e202413182. 39045883 10.1002/anie.202413182

[26] C. Li , K. Zhang , and W. Zhao , “Catalytic Contra‐Thermodynamic Isomerization–Asymmetric Hydroboration of Alkenyl Alcohols and Amines,” ACS Catalysis 14 (2024): 5458–5468.

[27] I. Massad , R. Suresh , L. Segura , and I. Marek , “Stereoselective Synthesis through Remote Functionalization,” Nature Synthesis 1 (2022): 37–48.

[28] Y. Yao , J. Sun , J. Li , and W. Zhao , “Catalytic Asymmetric Isomerization/Hydroboration of Silyl Enol Ethers,” Chemical Science 16 (2025): 20275–20285. 41050588 10.1039/d5sc04819b PMC12495312

[29] S. Kanno , F. Kakiuchi , and T. Kochi , “Palladium‐Catalyzed Remote Diborylative Cyclization of Dienes with Diborons via Chain Walking,” Journal of the American Chemical Society 143 (2021): 19275–19281. 34695350 10.1021/jacs.1c09705

[30] R. I. Pugh , E. Drent , and P. G. Pringle , “Tandem Isomerisation‐Carbonylation Catalysis: Highly Active Palladium(ii) Catalysts for the Selective Methoxycarbonylation of Internal Alkenes to Linear Esters,” Chemical Communications 1 (2001): 1476–1477.

[31] C. J. Rodriguez , D. F. Foster , G. R. Eastham , and D. J. Cole‐Hamilton , “Highly Selective Formation of Linear Esters from Terminal and Internal Alkenes Catalysed by Palladium Complexes of Bis‐(di‐Tert‐Butylphosphinomethyl)benzene,” Chemical Communications 4 (2004): 1720–1721. 10.1039/b404783d 15278154

[32] F. Lehmann , H. W. Wegener , N. Brede , and T. Seidensticker , “Combining Partial Hydrogenation of Soybean‐Derived Biodiesel and Methoxycarbonylation Using a Single Palladium Source,” European Journal of Lipid Science and Technology 127 (2025): e70073.

[33] M. R. L. Furst , R. L. Goff , D. Quinzler , S. Mecking , C. H. Botting , and D. J. Cole‐Hamilton , “Polymer Precursors from Catalytic Reactions of Natural Oils,” Green Chemistry 14 (2012): 472–477.

[34] V. Goldbach , L. Falivene , L. Caporaso , L. Cavallo , and S. Mecking , “Single‐Step Access to Long‐Chain α,ω‐Dicarboxylic Acids by Isomerizing Hydroxycarbonylation of Unsaturated Fatty Acids,” ACS Catalysis 6 (2016): 8229–8238.

[35] N. Herrmann , K. Köhnke , and T. Seidensticker , “Selective Product Crystallization for Concurrent Product Separation and Catalyst Recycling in the Isomerizing Methoxycarbonylation of Methyl Oleate,” ACS Sustainable Chemistry & Engineering 8 (2020): 10633–10638.

[36] F. Stempfle , D. Quinzler , I. Heckler , and S. Mecking , “Long‐Chain Linear C19 and C23 Monomers and Polycondensates from Unsaturated Fatty Acid Esters,” Macromolecules 44 (2011): 4159–4166.

[37] J. D. Nobbs , C. H. Low , L. P. Stubbs , C. Wang , E. Drent , and M. van Meurs , “Isomerizing Methoxycarbonylation of Alkenes to Esters Using a Bis(phosphorinone)xylene Palladium Catalyst,” Organometallics 36 (2017): 391–398.

[38] C. Jiménez‐Rodriguez , G. R. Eastham , and D. J. Cole‐Hamilton , “Dicarboxylic Acid Esters From the Carbonylation of Unsaturated Esters Under Mild Conditions,” Inorganic Chemistry Communications 8 (2005): 878–881.

[39] J. T. Christl , P. Roesle , F. Stempfle , et al., “Promotion of Selective Pathways in Isomerizing Functionalization of Plant Oils by Rigid Framework Substituents,” ChemSusChem 7 (2014): 3491–3495. 25314333 10.1002/cssc.201402441

[40] D. Quinzler and S. Mecking , “Linear Semicrystalline Polyesters from Fatty Acids by Complete Feedstock Molecule Utilization,” Angewandte Chemie International Edition 49 (2010): 4306–4308. 20455236 10.1002/anie.201001510

[41] F. Stempfle , B. S. Ritter , R. Mülhaupt , and S. Mecking , “Long‐Chain Aliphatic Polyesters from Plant Oils for Injection Molding, Film Extrusion and Electrospinning,” Green Chemistry 16 (2014): 2008–2014.

[42] P. Roesle , L. Caporaso , M. Schnitte , V. Goldbach , L. Cavallo , and S. Mecking , “A Comprehensive Mechanistic Picture of the Isomerizing Alkoxycarbonylation of Plant Oils,” Journal of the American Chemical Society 136 (2014): 16871–16881. 25415929 10.1021/ja508447d

[43] M. Gaydou , T. Moragas , F. Juliá‐Hernández , and R. Martin , “Site‐Selective Catalytic Carboxylation of Unsaturated Hydrocarbons with CO _2_ and Water,” Journal of the American Chemical Society 139 (2017): 12161–12164. 28814076 10.1021/jacs.7b07637

[44] M. Vilches‐Herrera , L. Domke , and A. Börner , “Isomerization–Hydroformylation Tandem Reactions,” ACS Catalysis 4 (2014): 1706–1724.

[45] R. Zhang , X. Yan , C. Chen , et al., “Integration of Milstein Ru–PNN and Rh–Tribi/Tetrabi for Isomerization Linear Selective Hydroformylation of Far Internal Alkenes,” The Journal of Organic Chemistry 87 (2022): 16941–16946. 36473047 10.1021/acs.joc.2c02423

[46] C. Cai , S. Yu , G. Liu , X. Zhang , and X. Zhang , “Highly Regioselective Isomerization–Hydroformylation of Internal Olefins Catalyzed by Rhodium/Tetraphosphine Complexes,” Advanced Synthesis & Catalysis 353 (2011): 2665–2670.

[47] R. Zhang , X. Yan , S. T. Bai , et al., “Examination of Milstein Ru‐PNN and Rh‐Tribi/Tetrabi Dual Metal Catalyst for Isomerization‐Linear‐Hydroformylation of C4 Raffinate and Internal Olefins,” Green Synthesis and Catalysis 3 (2022): 40–45.

[48] S. Tang , Y. Jiang , J. Yi , et al., “Highly Regioselective Homogeneous Isomerization‐Hydroformylation of 2‐Butene with Water‐ and Air‐Stable Phosphoramidite Bidentate Ligand,” Molecular Catalysis 508 (2021): 111598.

[49] A. Behr , D. Obst , C. Schulte , and T. Schosser , “Highly Selective Tandem Isomerization‐Hydroformylation Reaction of Trans‐4‐Octene to n‐Nonanal with Rhodium‐BIPHEPHOS Catalysis,” Journal of Molecular Catalysis A 206 (2003): 179–184.

[50] W. Ren , J. Huang , J. Ou , Y. Shi , and Y. Shi , “Pd‐Catalyzed Regioselective Isomerization‐Hydroformylation of Internal Olefins with HCO _2_ H and *N* ‐Formylsaccharin,” Chinese Journal of Chemistry 43 (2025): 3295–3301.

[51] B. Zhang , C. Kubis , and R. Franke , “Hydroformylation Catalyzed by Unmodified Cobalt Carbonyl Under Mild Conditions,” Science 377 (2022): 1223–1227. 36074860 10.1126/science.abm4465

[52] L. L. Goanvic , J. L. Couturier , J. L. Dubois , and J. F. Carpentier , “Ruthenium‐Catalyzed Hydroformylation of the Functional Unsaturated Fatty Nitrile 10‐Undecenitrile,” Journal of Molecular Catalysis A 417 (2016): 116–121.

[53] C. Breschi , L. Piparo , P. Pertici , A. M. Caporusso , and G. Vitulli , “η6‐Cyclohepta‐1,3,5‐Triene)(η4‐Cycloocta‐1,5‐Diene)iron(0) Complex as Attractive Precursor in Catalysis,” Journal of Organometallic Chemistry 607 (2000): 57–63.

[54] A. Behr , D. Obst , and A. Westfechtel , “Isomerizing hydroformylation of fatty acid esters: Formation of ω‐aldehydes,” European Journal of Lipid Science and Technology 107 (2005): 213–219.

[55] Y. Yuki , K. Takahashi , Y. Tanaka , and K. Nozaki , “Tandem Isomerization/Hydroformylation/Hydrogenation of Internal Alkenes to n‐Alcohols Using Rh/Ru Dual‐or Ternary‐Catalyst Systems,” Journal of the American Chemical Society 135 (2013): 17393–17400. 24191719 10.1021/ja407523j

[56] S. Pandey and S. H. Chikkali , “Highly Regioselective Isomerizing Hydroformylation of Long‐Chain Internal Olefins Catalyzed by a Rhodium Bis(Phosphite) Complex,” ChemCatChem 7(2015):3468–3471.

[57] T. Gaide , J. Bianga , K. Schlipköter , A. Behr , and A. J. Vorholt , “Linear Selective Isomerization/Hydroformylation of Unsaturated Fatty Acid Methyl Esters: A Bimetallic Approach,” ACS Catalysis 7 (2017): 4163–4171.

[58] S. Pandey , D. R. Shinde , and S. H. Chikkali , “Isomerizing Hydroformylation of Cashew Nut Shell Liquid,” ChemCatChem 9(2017):3997–4004.

[59] L. Wu , I. Fleischer , R. Jackstell , I. Profir , R. Franke , and M. Beller , “Ruthenium‐Catalyzed Hydroformylation/Reduction of Olefins to Alcohols: Extending the Scope to Internal Alkenes,” Journal of the American Chemical Society 135 (2013): 14306–14312. 23987582 10.1021/ja4060977

[60] F. M. S. Rodrigues , P. K. Kucmierczyk , M. Pineiro , et al., “Dual Rh−Ru Catalysts for Reductive Hydroformylation of Olefins to Alcohols,” ChemSusChem 11 (2018): 2310–2314. 29874413 10.1002/cssc.201800488

[61] X. Tang , L. Gan , X. Zhang , and Z. Huang , “ *n* ‐Alkanes to *n* ‐alcohols: Formal primary C—H bond hydroxymethylation via quadruple relay catalysis,” Science Advances 6 (2020): eabc6688. 33219029 10.1126/sciadv.abc6688 PMC7679163

[62] A. Seayad , M. Ahmed , H. Klein , R. Jackstell , T. Gross , and M. Beller , “Internal Olefins to Linear Amines,” Science 297 (2002): 1676–1678. 12215640 10.1126/science.1074801

[63] G. Liu , K. Huang , B. Cao , et al., “Highly Regioselective Isomerization‐Hydroaminomethylation of Internal Olefins Catalyzed by Rh Complex with Tetrabi‐Type Phosphorus Ligands,” Organic Letters 14 (2012): 102–105. 22149490 10.1021/ol202848a

[64] M. Ahmed , R. P. J. Bronger , R. Jackstell , P. C. J. Kamer , P. W. N. M. Van Leeuwen , and M. Beller , “Highly Selective Hydroaminomethylation of Internal Alkenes to Give Linear Amines,” Chemistry ‐ A European Journal 12 (2006): 8979–8988. 17013965 10.1002/chem.200600702

[65] G. Occhialini , V. Palani , and A. E. Wendlandt , “Catalytic, Contra‐Thermodynamic Positional Alkene Isomerization,” Journal of the American Chemical Society 144 (2022): 145–152. 34968044 10.1021/jacs.1c12043

[66] K. Zhao and R. R. Knowles , “Contra‐Thermodynamic Positional Isomerization of Olefins,” Journal of the American Chemical Society 144 (2022): 137–144. 34968043 10.1021/jacs.1c11681

[67] X. Li , J. Jin , P. Chen , and G. Liu , “Catalytic Remote Hydrohalogenation of Internal Alkenes,” Nature Chemistry 14 (2022): 425–432. 10.1038/s41557-021-00869-x 35102326

[68] J. Gao , M. Jiao , J. Ni , R. Yu , G. J. Cheng , and X. Fang , “Nickel‐Catalyzed Migratory Hydrocyanation of Internal Alkenes: Unexpected Diastereomeric‐Ligand‐Controlled Regiodivergence,” Angewandte Chemie International Edition 60 (2021): 1883–1890. 33021014 10.1002/anie.202011231

[69] X. Li , X. Yang , P. Chen , and G. Liu , “Palladium‐Catalyzed Remote Hydro‐Oxygenation of Internal Alkenes: An Efficient Access to Primary Alcohols,” Journal of the American Chemical Society 144 (2022): 22877–22883. 36508607 10.1021/jacs.2c11428

[70] N. Bai , X. Wang , Z. Wang , F. Liu , and Z. Q. Rong , “Redox‐Neutral Remote Amidation of Alkenyl Alcohols via Long‐Range Isomerization/Transformation,” Organic Chemistry Frontiers 9 (2022): 5942–5948.

[71] Q. Wang , H. Jung , D. Kim , and S. Chang , “Iridium‐Catalyzed Migratory Terminal C(Sp3)‐H Amidation of Heteroatom‐Substituted Internal Alkenes via Olefin Chain Walking,” Journal of the American Chemical Society 145 (2023): 24940–24951. 10.1021/jacs.3c09679 37906814

[72] G. E. Dobereiner , G. Erdogan , C. R. Larsen , D. B. Grotjahn , and R. R. Schrock , “A One‐Pot Tandem Olefin Isomerization/Metathesis‐Coupling (ISOMET) Reaction,” ACS Catalysis 4 (2014): 3069–3076.

[73] S. Z. Sun , M. Börjesson , R. Martin‐Montero , and R. Martin , “Site‐Selective Ni‐Catalyzed Reductive Coupling of α‐Haloboranes with Unactivated Olefins,” Journal of the American Chemical Society 140 (2018): 12765–12769. 30244574 10.1021/jacs.8b09425

[74] F. Zhou , J. Zhu , Y. Zhang , and S. Zhu , “NiH‐Catalyzed Reductive Relay Hydroalkylation: A Strategy for the Remote C(sp 3)−H Alkylation of Alkenes,” Angewandte Chemie 130 (2018): 4122–4126. 10.1002/anie.201712731 29460343

[75] Z. Y. Wang , J. H. Wan , G. Y. Wang , et al., “Terminal C(Sp3)–H Alkylation of Internal Alkenes via Ni/H‐Catalyzed Isomerization,” Tetrahedron Letters 59 (2018): 2302–2305.

[76] I. Marek , N. Chinkov , and A. Levin , “A Zirconium Promenade ‐ An Efficient Tool in Organic Synthesis,” Synlett (2006): 0501–0514.

[77] L. Mola , M. Sidera , and S. P. Fletcher , “Asymmetric Remote C‐H Functionalization: Use of Internal Olefins in Tandem Hydrometallation‐Isomerization‐Asymmetric Conjugate Addition Sequences,” Australian Journal of Chemistry 68 (2015): 401–403.

[78] K. H. N. Tang , R. Tokutake , M. Ito , and T. Shibata , “Ir‐Catalyzed Distal Branch‐Selective Hydroarylation of Unactivated Internal Alkenes with Benzanilides via C‐H Activation along with Consecutive Isomerization,” Organic Letters 25 (2023): 5197–5202. 37427870 10.1021/acs.orglett.3c01619

[79] H. Takahashi and T. Shibata , “Iridium‐Catalyzed Linear‐Selective sp3 C−H Alkylation of N‐Methylamides Using Alkenes Enabled by Diphosphite Ligands,” Chemistry – A European Journal (2026): e70913. 41870077 10.1002/chem.70913

[80] K. H. N. Tang , K. Uchida , K. Nishihara , M. Ito , and T. Shibata , “Ir‐Catalyzed Remote Functionalization by the Combination of Deconjugative Chain‐Walking and C‐H Activation Using a Transient Directing Group,” Organic Letters 24 (2022): 1313–1317. 35139636 10.1021/acs.orglett.1c04321

[81] A. J. Borah and Z. Shi , “Rhodium‐Catalyzed, Remote Terminal Hydroarylation of Activated Olefins through a Long‐Range Deconjugative Isomerization,” Journal of the American Chemical Society 140 (2018): 6062–6066. 29727177 10.1021/jacs.8b03560

[82] D. Heng , H. Chen , X. He , et al., “Synergistic Dinuclear Rhodium Induced Rhodium‐Walking Enabling Alkene Terminal Arylation: A Theoretical Study,” ACS Catalysis 11 (2021): 3975–3987.

[83] S. Imran , Y. Liu , Y. Li , Y. Shui , C. H. Yan , and H. M. Sun , “Nickel‐Catalyzed Tandem Isomerization/Anti‐Markovnikov Hydroarylation of Unactivated Internal Alkenes with Heteroarenes,” Organic Chemistry Frontiers 10 (2023): 1361–1367.

[84] Y. He , B. Han , and S. Zhu , “Terminal‐Selective C(Sp3)‐H Arylation: NiH‐Catalyzed Remote Hydroarylation of Unactivated Internal Olefins,” Organometallics 40 (2021): 2253–2264.

[85] M. L. Cano , D. M. Hamilton , and T. B. Thomason , Production of 1‐Alkenes From Mixed Olefin Streams Using Catalytic Distillation, 2005, WO 2005/035469 A1.

